# Prevent cervical cancer by screening with reliable human papillomavirus detection and genotyping

**DOI:** 10.1002/cam4.9

**Published:** 2012-07-05

**Authors:** Shichao Ge, Bo Gong, Xushan Cai, Xiaoer Yang, Xiaowei Gan, Xinghai Tong, Haichuan Li, Meijuan Zhu, Fengyun Yang, Hongrong Zhou, Guofan Hong

**Affiliations:** 1 State Key Laboratory of Molecular Biology, Institute of Biochemistry and Cell Biology, Shanghai Institutes for Biological Sciences, Chinese Academy of SciencesShanghai, China; 2 Department of Pediatrics, Changning District Maternal and Children Health Care HospitalShanghai, China; 3 Department of Pediatrics, Jiading District Maternal and Children Health Care HospitalShanghai, China

**Keywords:** Cervical cancer, HPV, prevention, screen

## Abstract

The incidence of cervical cancer is expected to rise sharply in China. A reliable routine human papillomavirus (HPV) detection and genotyping test to be supplemented by the limited Papanicolaou cytology facilities is urgently needed to help identify the patients with cervical precancer for preventive interventions. To this end, we evaluated a nested polymerase chain reaction (PCR) protocol for detection of HPV L1 gene DNA in cervicovaginal cells. The PCR amplicons were genotyped by direct DNA sequencing. In parallel, split samples were subjected to a Digene HC2 HPV test which has been widely used for “cervical cancer risk” screen. Of the 1826 specimens, 1655 contained sufficient materials for analysis and 657 were truly negative. PCR/DNA sequencing showed 674 infected by a single high-risk HPV, 188 by a single low-risk HPV, and 136 by multiple HPV genotypes with up to five HPV genotypes in one specimen. In comparison, the HC2 test classified 713 specimens as infected by high-risk HPV, and 942 as negative for HPV infections. The high-risk HC2 test correctly detected 388 (57.6%) of the 674 high-risk HPV isolates in clinical specimens, mislabeled 88 (46.8%) of the 188 low-risk HPV isolates as high-risk genotypes, and classified 180 (27.4%) of the 657 “true-negative” samples as being infected by high-risk HPV. It was found to cross-react with 20 low-risk HPV genotypes. We conclude that nested PCR detection of HPV followed by short target DNA sequencing can be used for screening and genotyping to formulate a paradigm in clinical management of HPV-related disorders in a rapidly developing economy.

## Introduction

Since opening up to the outside world, China has undergone tremendous changes both economically and socially. Many sexually transmitted diseases which were fairly rare some 30 years ago are now common occurrences [1]. Health authorities are particularly concerned about the emerging increase in the number of patients suffering from cervical cancer. The Chinese government has implemented a pilot project for free cervical cancer screening [2]. A national plan is therefore urgently needed to prevent an impending rise in the incidence of cervical cancer which is known to be initiated by persistent human papillomavirus (HPV) infections [3], but takes decades to develop [4].

Cervical cancer is a major fatal malignancy among women, causing about 275,000 deaths annually worldwide, most in developing countries [5]. It is also a highly preventable cancer if detected at its precancerous stages and treated by ablative procedures. In the United States, the widespread use of Papanicolaou (Pap) smear screening for detection followed by treatment of the precancerous lesions reduced the incidence of cervical cancer from 44 in 100,000 women in 1947 to 8.8 in 1970 [6]. The current age-standardized mortality rate is 1.7 per 100,000 [5]. Cervical cancer is primarily a disease among unscreened or rarely screened women [7].

In resource-constrained settings, community outreach, education, and advocacy are the key components in advancing cervical cancer prevention initiatives [8], and the demand for Pap smear screening invariably exceeds the existent quality cytopathology service capacity [9]. Alternatively, a molecular test for the presence of persistent HPV infection has been recommended for the initial cervical screening in place of the traditional morphological programs based on Pap cytology for cervical cancer prevention [10–12].

Various HPV DNA tests have been introduced to meet the need for effective cervical cancer prevention screening in China [13, 14] and in other countries [15–20]. However, these tests without a uniform standard frequently generate discordant results. For example, a widely marketed Digene HC2 high-risk HPV assay (Digene Corporation, Gaithersburg, MD) for the detection of 13 high-risk HPV genotypes in cervicovaginal samples is often used as the reference method to validate others [21, 22]. But when the HC2 assay was compared with another FDA-approved Cervista™ HPV test (Hologic/ThirdWave Technologies, Marborough, Massachusetts), the latter was found to generate two to four times more positive results than the former [23]. Either assay may have triaged an undetermined number of women to unnecessary work up [24]. In fact, more than 95% of the referrals based on an HC2-positive test result to colposcopic biopsy for cancer-diagnostic workup have been found to be excessive [25]. These unnecessary biopsy procedures are difficult to sustain by resource-constrained communities, let alone the cost associated with managing the adverse side effects of the needless 4-quadrant cervical biopsies. If an HPV DNA test is to be adopted as a routine procedure for cervical screen as proposed [10–12, 26], the analytical sensitivity and specificity of the test must be rigorously validated.

Since DNA sequencing is the generally accepted standard for molecular identification and genotyping of HPV [27], an HPV-positive test result validated with genotyping by DNA sequencing is beyond a reasonable doubt and is highly valuable for following persistent HPV infections. In this report, we recommend with supportive scientific evidence that a nested polymerase chain reaction (PCR) detection of HPV followed by short target DNA sequencing be used for screening and genotyping to formulate a paradigm in clinical management of HPV-related disorders in a rapidly developing country, like China. In comparison, we have demonstrated that the widely used Digene HC2 test generates a large number of false-negative and false-positive results which may lead to a substantial waste of the limited health care resources.

## Materials and Methods

This study was approved by the Institutional Review Boards of the Shanghai Institute of Biochemistry and Cell Biology (SIBCB), Shanghai Institutes for Biological Sciences, Chinese Academy of Sciences, Changning District Maternal and Children Health Care Hospital (CMCHCH), and Jiading District Maternal and Children Health Care Hospital (JMCHCH).

The clinical samples used for this study were from 1826 women visiting the gynecology clinics of the two above-referenced hospitals. The median age of the patients was 39.8 years (range from 20 to 65 years old). CMCHCH is a hospital in a peripheral district and JMCHCH in the suburb of Shanghai. Both hospitals serve an economically rapidly developing community. The patients included in this study were those who visited the clinics for a variety of minor gynecologic symptomatic disorders other than neoplastic diseases. The majority of the patients did not have prior regular gynecologic care, but were generally aware of the value of a “screen test” for cervical cancer prevention, and were willing to pay for a DNA “cancer screen” approved by the U.S. Food and Drug Administration (FDA) at the cost of about U.S. $50 per test to determine a cervical cancer risk. They were not willing to pay for an additional Pap cytology screen.

According to the protocols of practice, the cervicovaginal cells at the transformation zone of the uterine cervix were collected by a gynecologist or a trained gynecologist assistant with a standard cytobrush (with spatula), and suspended in PreservCyt® or Surepath® fixative for routine Digene HC2 assays (Digene Corporation, Gaithersburg, Maryland) to detect high-risk HPV DNA. With the patient's informed consent, an aliquot of the collected cell suspension from each double-blind number-coded sample was used for the HPV DNA nested PCR amplification followed by HPV genotyping by DNA sequencing if found to be HPV positive.

The HC2 test was performed in the pathology laboratories at CMCHCH and JMCHCH according to instructions provided by Digene Corporation. Nested PCR HPV DNA detection followed by genotyping with short target DNA sequencing, using the MY09/MY11 degenerate primer pair for primary PCR and the GP6/MY11 primer pair for nested PCR, was performed at the SIBCB laboratory according to a low temperature PCR program as reported in the literature [28]. Each nested PCR amplicon of about ∼190 bp in size was confirmed by direct automated DNA sequencing, using GP6 nucleotide as the sequencing primer. The genotype-specific sequence was validated through online BLAST algorithms. A 100% identity match with an HPV DNA sequence for at least 40 bases in this hypervariable L1 gene region categorized in the GenBank database determined the HPV genotype.

When more than one HPV genotype was present in a sample, the routine DNA sequencing on the GP6/MY11 nested PCR products was unable to generate a readable base-calling electropherogram because more than one HPV DNA template had been amplified by the degenerate and consensus general PCR primers. In order to determine accurately the prevalence of genotypes involved in these specimens, the ∼450 bp primary PCR products of 50 randomly selected samples with multiple HPV infections were reamplified, each by 24 individual genotype-specific nested PCR primer pairs listed in Table 1. All positive genotype-specific nested PCR amplicons, ranging from 150 to 345 bp in size, were individually sequenced using one of the genotype-specific primers as the sequencing primer. These 24 pairs of genotype-specific nested PCR primers were designed using “Software Primer3” as previously reported [29], targeting a hypervariable segment of the MY09/MY11 amplicon of the 13 generally recognized high-risk HPV genotypes and the 11 commonly encountered “low-risk” HPV genotypes observed in China based on prior experience.

**Table 1:** Type-specific primers for detection of HPV types in the multiple HPV-infected specimen.^a^

HPV types	Forward primers (5′→3′)	Reverse primers (5′→3′)	Size of product (bp)
HPV-16	TACCTACGACATGGGGAGGA	GCAATTGCCTGGGATGTTAC	194
HPV-18	TGGTGTTTGCTGGCATAATC	GCAGCATCCTTTTGACAGGT	339
HPV-31	AATATGTCTGTTTGTGCTGCAA	CTGAGGGAGGTGTGGTCAAT	214
HPV-33	TGGGGCAATCAGGTATTTGT	GGGGTCTTCCTTTCCTTTG	345
HPV-35	TGTCTGTGTGTTCTGCTGTGTC	GTTTTGGTGCACTGGGTTTT	282
HPV-39	GGCACGTGGAGGAGTATGAT	TCTTTCTTTTCAGGTGCTGGA	223
HPV-45	ACACAAAATCCTGTGCCAAA	TCCTGCTTTTCTGGAGGTGT	278
HPV-51	GGCATGGGGAAGAGTATGAA	GATCTGGCTTAGCCTGTGGA	225
HPV-52	ATGTTGGGGCAATCATTGT	TGTGTCCTCCAAAGATGCAG	280
HPV-56	CATTTGCTGGGGTAATCAAT	CGGGGATAACCCAATATTCC	256
HPV-58	TTGCTGGGGCAATCAGTTAT	CCTTTTCTTTAGGGGGTGCT	341
HPV-59	TATGCCAGACATGTGGAGGA	GCGGTGTCCTTTTGACAAGT	212
HPV-68	GGATACCACTCGCAGTACCAA	AGGGGCAACACCAAAATTC	226
HPV-6	ACATGCGTCATGTGGAAGAG	AGGTAATGGCCTGTGACTGC	195
HPV-11	GCCATGTGGAGGAGTTTGAT	AGGTGTGGGTTTCTGACAGG	206
HPV-40	CCCACACCAACCCCATATAA	CAGGCAATAGCCTTGTTGGT	236
HPV-53	ATGACTCTTTCCGCAACCAC	AACAGGAGGCGACAAACCTA	204
HPV-54	TACAGCAACCTCGCAGGATA	CCAAATTCCAGTCCTCCAA	174
HPV-62	AGGGAATTTTTGCGACACAC	GCCCGAGACTGCAAATAGTG	194
HPV-66	GATGCACGTGAAATCAATCAA	GGGACAATCCAATGTTCCAA	157
HPV-69	CACAATCTGCATCTGCCACT	AGGCAAGGTAAGGCCAAAAT	185
HPV-70	AAACGGCCATACCTGCTGTA	GGAGCATCCTTTTGACATGC	256
HPV-73	TGGAAGAGTGGAATTTTGGTC	CATCCCAAAAGGATAGCTTGG	150
HPV-81	ATTTCTGCGCCATACAGAGG	GGTAATGGCCCGAGACTGTA	196

a Specimen infected by more than one HPV genotype.

A β-globin primer pair for human genomic DNA amplification was used to control specimen adequacy. Specimens negative for HPV DNA with no companion β-globin gene amplification were excluded as insufficient. However, specimens found to be positive for HPV DNA by nested PCR and confirmed by DNA sequencing were considered sufficient even when no β-globin gene amplification was observed.

## Results

All visualized GP6/MY11 primer-defined nested PCR amplicons were confirmed by direct automated fluorescent dye-terminator Sanger method to contain HPV DNA sequences. Of the 1826 liquid-based cervicovaginal specimens tested by both nested PCR and the HC2 kit on split samples in parallel, 171 were found to be negative for β-globin gene amplification and negative for HPV DNA by either nested PCR or by the HC2 kit, and were excluded as insufficient for evaluation. Of the remaining 1655 specimens tested by both methods, the PCR/DNA sequencing protocol showed 674 infected by a single high-risk HPV, 188 by a single low-risk HPV, and 136 by multiple HPV genotypes, 657 samples were negative by HPV DNA PCR, and positive for β-globin gene amplification, and therefore accepted as “true negative” for HPV infection (Table 2).

**Table 2:** Comparison between the results derived by nested PCR/DNA sequencing and HC2 testing assays

	Nested PCR/DNA sequencing assay results				
	
HC2 results	High risk^a^	Low risk^b^	Mul-infection^c^	No infection^d^	Total
Positive	388	88	57	180	713
Negative	286	100	79	477	942
**Total**	**674**	**188**	**136**	**657**	**1655**

a Including 13 “high-risk” types targeted by HC2 assay.

b Indicating those untargeted by the HC2 assay.

c Specimen contained more than one HPV genotype.

d β-globin PCR positive, and both HPV primary PCR and nested PCR negative.

In comparison, the HC2 test classified 713 specimens as infected by high-risk HPV, and 942 as negative for HPV infections. Correlation of the results generated by these two methods on split samples showed that the HC2 test detected 388 (57.6%) of the 674 high-risk HPV isolates in clinical specimens, mislabeled 88 (46.8%) of the 188 low-risk HPV isolates as high-risk genotypes, and classified 180 (27.4%) of the 657 “true-negative” samples as being infected by high-risk HPV (Table 2). The sensitivity for the detection of single high-risk HPV infections using the HC2 test kit appeared to be genotype-dependent, ranging from 38.9% to 100%, with a detection rate of HPV-16 as low as 44.2% (Table 3). Of the commonly encountered HPV genotypes, 20 low-risk HPV genotypes were found to cross-react with the HC2 high-risk test. Cross-reactions with HPV-6, -11, -53, -54, -66, and -81 generated 66 (75%) of the 88 false-positive high-risk HPV HC2 tests (Table 4).

**Table 3:** Sensitivity of HC2 assay in detection of high-risk HPV DNA compared with nested PCR/DNA sequencing assay

Nested PCR/DNA sequencing results		Digene HC2 results		
		
High-risk types^a^	Cases	Positive	Negative	Sensitivity (%)
HPV-16	360	159	201	44.2
HPV-18	35	22	13	62.9
HPV-31	14	12	2	85.7
HPV-33	51	33	18	64.7
HPV-35	17	14	3	82.4
HPV-39	7	7	0	100
HPV-45	3	2	1	66.7
HPV-51	2	2	0	100
HPV-52	46	37	9	80.4
HPV-56	10	8	2	80
HPV-58	76	57	19	75
HPV-59	18	7	11	38.9
HPV-68	35	28	7	80
**Total**	**674**	**388**	**286**	**57.6**

a Indicating 13 high-risk types targeted by HC2 assay.

**Table 4:** Cross-reactivity of the HC2 testing in HPV detection determined by nested PCR/DNA sequencing assay

Nested PCR/DNA sequencing results		Digene HC2 results		
		
HPV types^a^	Cases	Positive	Negative	Cross-reactivity (%)
HPV-6	28	9	19	32.1
HPV-11	27	10	17	37
HPV-13	1	1	0	100
HPV-32	2	1	1	50
HPV-40	5	1	4	20
HPV-42	1	1	0	100
HPV-43	1	1	0	100
HPV-53	15	12	3	80
HPV-54	21	10	11	47.6
HPV-55	1	1	0	100
HPV-61	4	2	2	50
HPV-62	10	3	7	30
HPV-66	10	8	2	80
HPV-67	1	1	0	100
HPV-69	2	1	1	50
HPV-70	3	2	1	66.7
HPV-71	1	0	1	0
HPV-73	1	1	0	100
HPV-81	38	7	21	44.7
HPV-82	1	1	0	100
HPV-83	1	0	1	0
HPV-84	13	5	8	38.5
HPV-87	1	0	1	0
**Total**	**188**	**8**8	**100**	**46.8**

a Indicating HPV types untargeted by HC2 assay.

Of the 50 samples infected by more than one HPV genotype selected for individual genotype-specific nested PCR amplifications, 34 (68%) samples were found to contain two HPV genotypes, 11 (22%) samples three HPV genotypes, and four (8%) samples four HPV genotypes. One (2%) sample contained five HPV genotypes (Table 5).

**Table 5:** HPV genotypes detected in specimen with multiple HPV infections^a^

Nested PCR/DNA sequencing results			Digene HC2 results	
			
HPV types	Cases	Prevalence (%)	Positive	Negative
HPV-16,6	4	8	3	1
HPV-16,11	1	2	0	1
HPV-16,33	1	2	0	1
HPV-16,39	1	2	1	0
HPV-16,45	1	2	1	0
HPV-16,52	7	14	5	2
HPV-16,53	1	2	1	0
HPV-16,58	2	4	2	0
HPV-16,59	1	2	0	1
HPV-16,62	2	4	1	1
HPV-16,66	2	4	1	1
HPV-16,68	1	2	1	0
HPV-16,81	1	2	1	0
HPV-18,52	2	4	2	0
HPV-31,58	1	2	1	0
HPV-35,6	1	2	1	0
HPV-45,58	1	2	1	0
HPV-52,6	2	4	1	1
HPV-52,58	1	2	1	0
HPV-56,81	1	2	1	0
**Two types**	**34**	**68**	**25**	**9**
HPV-16,6,53	1	2	0	1
HPV-16,18,66	1	2	0	1
HPV-16,31,62	1	2	0	1
HPV-16,31,66	1	2	1	0
HPV-16,31,81	1	2	1	0
HPV-16,39,62	1	2	1	0
HPV-16,52,58	1	2	0	1
HPV-16,52,62	1	2	1	0
HPV-16,52,68	1	2	1	0
HPV-16,59,62	1	2	0	1
HPV-52,54,81	1	2	1	0
**Three types**	**11**	**22**	**6**	**5**
HPV-16,6,33,52	1	2	1	0
HPV-16,6,59,66	1	2	1	0
HPV-16,31,53,81	1	2	1	0
HPV-31,6,52,66	1	2	1	0
**Four types**	**4**	**8**	**4**	**0**
HPV-16,52,56,58,81	1	2	0	1
**Five types**	**1**	**2**	**0**	**1**
**Total**	**50**	**100**	**35**	**15**

a Specimen infected by more than one HPV genotype.

**Figure 1:**
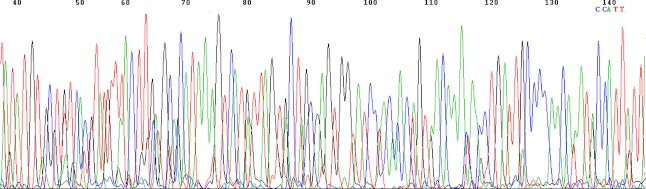
Sample of multiple HPV infections. The electropherogram generated by an ABI 3130 Genetic Analyzer with integrated computer (Applied Biosystems, Foster City, CA) failed to yield a readable sequence for HPV genotyping due to multiple overlapping DNA sequences in this base-calling segment. However, in majority of cases, two conserved HPV sequences, AAA and CCATT as indicated, can be identified to show that the unreadable electropherogram represents a mixture of HPV DNA sequences.

**Figure 2:**
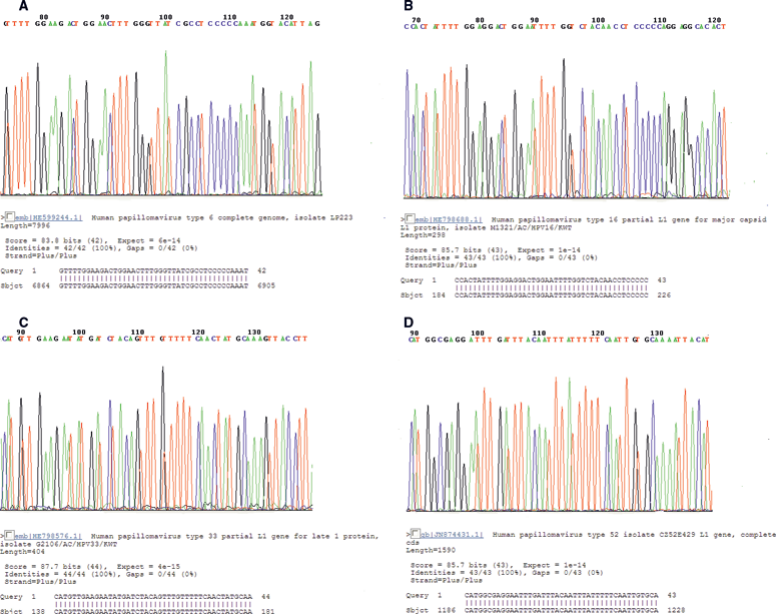
Four HPV genotypes found in one sample. The MY09/MY11 primary PCR product from the case shown in Figure 1 was reamplified individually by 24 pairs of genotype-specific nested PCR primers (Table 1). The four positive genotype-specific nested PCR amplicons were validated by DNA sequencing to be those of HPV-6 (A), HPV-16 (B), HPV-33 (C), and HPV-52 (D), respectively, by online BLAST alignment analyses.

Base-calling invariably failed to resolve mixed DNA sequences generated from more than one nested PCR amplicon in a single Sanger reaction (Fig. 1). However, after the individual nested PCR amplifications with genotype-specific primers (Table 1), the type-specific nested PCR amplicons of the HPV genotypes in the sample were readily confirmed by direct DNA sequencing (Fig. 2 A–D). All 50 mixed HPV-infected samples which were tested by specific nested PCR harbored at least one of the 13 high-risk HPV genotypes. In fact, 41 (82%) of the 50 samples infected by multiple HPV genotypes were found to contain an HPV-16 in addition to various other companion genotypes (Table 5).

## Discussion

Although cervical cancers are initiated by persistent infection of high-risk HPV, less than 10% of new HPV infections result in persistent HPV infections and precancerous pathologies, typically within 5–10 years. Invasive cancer only develops in a very small percentage of women with precancerous cellular changes over one to three decades [10]. A rapidly developing country like China needs a reliable approach to identify the sexually active women with precancer for ablative procedures to prevent life-threatening invasive cervical cancers that are expected to develop in the coming decades.

In developed countries, women with cervical precancer usually have a positive Pap cytology screening result of high-grade squamous intraepithelial lesion (HSIL), then are referred to colposcopic biopsy for a histological diagnosis of cervical intraepithelial neoplasia grade 2 or grade 3 (CIN2 or CIN3) before an ablative procedure is performed. Medical practice guidelines are against unnecessary colposcopic biopsies and needless traumatic surgical procedures which may have undesirable health consequences [30].

In developing countries, quality cytology screen, colposcopic biopsies and histopathological diagnostic services are not readily available. The HC2 test kit or its cheaper version has been recommended for cancer screening in place of Pap cytology in China [14, 31]. The major concern with such an HPV screening program is how to appropriately manage the large number of women with HPV-positive specimens [32], who have no cytological evidence of cervical precancer or cancer. The latter problem is further augmented by various HPV DNA tests generating discordant results in positivity and genotyping [13–20]. The limited health care resources available cannot sustain concomitant cervical screens with both HPV testing and Pap smear cytology for every woman at risk of developing cervical precancer. The only practical screen for cervical cancer prevention is to adopt a reliable HPV test for primary cervical screening to be supplemented by a traditional Pap cytology as needed.

A clinically useful HPV nucleic acid test must be able to reliably answer two questions: (1) Is there HPV DNA in the specimen? (2) What is the genotype of the HPV detected?

Since persistent HPV infection is a necessary factor for cervical cancer development, a truly negative HPV test result practically rules out the presence of cervical precancer or cancer in the specimen. As the pathology in persistent HPV infection advances to precancer and cancer, the viral load per abnormal cell tends to decrease while the HPV-rich koilocytes are progressively replaced by dysplastic cells with a higher nuclear/cytoplasmic ratio in the epithelum [33–35]. The technology of nested PCR detection followed by genotyping with direct DNA sequencing provides the required analytical sensitivity and specificity of a reliable HPV test. The risk of developing cancer is greatest in women positive for the same genotype of HPV on repeated testing [36, 37]. How to integrate such an analytically reliable molecular test into a system supplemented by cytopathology-based cervical screen for cervical cancer prevention must be determined by medical and scientific professionals with primary concern about improvement of women's health care under various resource settings rather than by business agenda of the industry.

The present study was performed using DNA sequencing as the gold standard for HPV screening [38], revealing that the widely used commercial Digene HC2 test kits, which are being used as the “cancer-screening test” in China, correctly detected merely 57.6% of the 674 high-risk HPV isolates in clinical specimens. That means 42.4% of the clinical specimens infected by a high-risk (or carcinogenic) HPV were missed by the HC2 test. These 42.4% false-negative high-risk HPV reports may deprive some patients with cervical precancer of the benefits of having further cytological evaluation which may be necessary for detection of HSILs and CIN2/3 that can be timely treated for cancer prevention. It implies that the HC2 test has a very low negative predictive value (npv) and cannot be relied upon as the “lone” cervical screen for cancer prevention, as the traditional Pap smear can. The positive predictive value (ppv) of Digene HC2 HPV testing without a companion high-grade cytology Pap smear for precancer among women 50 years and older was also very low [39]. The HPV DNA sequencing has a high npv in clinical practice, if HPV DNA test without a companion Pap smear is used for cervical screen and is a highly sensitive and specific HPV genotyping method. Using an HPV DNA test in guiding clinical practice exploits its high npv, and does not depend on its low ppv in predicting a histopathology of CIN2/3 for patient management.

At the analytical level, we confirm that the high-risk probe B cocktail of the Digene HC2 kit cross-reacts with the sequences of HPV-6, -11, -13, -32, -40, -42, -43, -53, -54, -55, -61, -62, -66, -67, -69, -70, -73, -81, -82, and -84. Cross-reaction causes 46.8% of the untargeted low-risk HPV genotypes to be mislabeled as high-risk HPV, which may lead to further unnecessary confusion. Compared with a nested PCR/DNA sequencing technology, the HC2 kit detects only 57.6% of the single high-risk HPV and 44.2% of HPV-16 infections. Since it cannot perform genotyping, the HC2 test does not offer the needed information for follow-up of persistent HPV infections.

Nested PCR is a recognized highly sensitive technology for HPV DNA detection and for preparing a template for DNA sequencing. Over 50% of the cervical HPV infections depend on a nested PCR DNA amplification for detection and subsequent validation [28]. The Digene HPV HC2 test is approved by the FDA as a DNA assay adjunctive to Pap cytology screening [40]. If used as a primary independent screening tool, according to a study of the U.S. National Cancer Institute, the sensitivity of the HC2 test to detect CIN3 or lesions higher than CIN3 has been calculated to be 96.3% (95% confidence interval [CI] = 91.6–98.8) [41]. The risk of missing an average of 3.7% preventable or curable cervical cancers would not be knowingly acceptable to the informed patients who are willing to finance their own health care in a rapidly developing China. In our series, 9.4% (171/1826) of the specimens collected at the women's health clinics are found to contain insufficient materials for evaluation due to absence the of β-globin gene amplification. These insufficient samples are routinely classified as HPV negative by the HC2 test. Such misclassification may further increase the false-negative rate with potential serious clinical outcomes if HC2 test is used as the only primary screening tool for detection of cervical precancer and cancer.

We designed a set of specific PCR primers for the 24 commonly encountered HPV genotypes to analyze samples infected with multiple HPV genotypes. For the samples containing more than one HPV type, the routine DNA sequencing was unable to generate a readable base-calling electropherogram as there was more than one type of PCR product presenting in PCR amplification mixtures. Theoretically, there exist variants in one genotype, so some of these genotype-specific primers listed may as well be “variant-specific”.

Our understanding of the clinical significance of multiple HPV infections is incomplete. For the first time, we have introduced a protocol of using 24 individual-specific nested PCR amplifications for each specimen containing more than one HPV for final genotyping by DNA sequencing. Preliminary observation indicates that each specimen with multiple HPV infections seems to contain at least one high-risk HPV genotype. Although this labor-intensive procedure requiring 24 nested PCRs for each sample with multiple HPV infections is cost-prohibitive in routine diagnostic practice, it may provide a valuable tool for further investigation of the evolvement of multiple HPV infections.
